# The Views and Experiences of Clinicians Sharing Medical Record Notes With Patients

**DOI:** 10.1001/jamanetworkopen.2020.1753

**Published:** 2020-03-27

**Authors:** Catherine M. DesRoches, Suzanne Leveille, Sigall K. Bell, Zhiyong J. Dong, Joann G. Elmore, Leonor Fernandez, Kendall Harcourt, Patricia Fitzgerald, Thomas H. Payne, Rebecca Stametz, Tom Delbanco, Jan Walker

**Affiliations:** 1 Harvard Medical School, Boston, Massachusetts; 2 Beth Israel Deaconess Medical Center, Boston, Massachusetts; 3 College of Nursing and Health Sciences, University of Massachusetts, Boston; 4 David Geffen School of Medicine, University of California, Los Angeles; 5 University of California, Los Angeles Fielding School of Public Health; 6 University of Washington, UW Medicine, Seattle; 7 Steele Institute for Health Innovation, Geisinger, Danville, Pennsylvania

## Abstract

**Importance:**

The 21st Century Cures Act of 2016 requires that patients be given electronic access to all the information in their electronic medical records. The regulations for implementation of this law give patients far easier access to information about their care, including the notes their clinicians write.

**Objective:**

To assess clinicians’ views and experiences with sharing clinical notes (open notes) with patients.

**Design, Setting, and Participants:**

Web-based survey study of physicians, advanced practice nurses, registered nurses, physician assistants, and therapists at 3 health systems in Boston, Massachusetts; Seattle, Washington; and rural Pennsylvania where notes have been shared across all outpatient specialties for at least 4 years. Participants were clinicians in hospital-based offices and community practices who had written at least 1 note opened by a patient in the year prior to the survey, which was administered from May 21, 2018, to August 31, 2018.

**Main Outcomes and Measures:**

Clinicians’ experiences with and perceptions of sharing clinical notes with patients.

**Results:**

Invitations were sent to 6064 clinicians; 1628 (27%) responded. Respondents were more likely than nonrespondents to be female (65% vs 55%) and to be younger (mean [SD] age, 42.1 [12.6] vs 44.9 [12.7] years). The majority of respondents were physicians (951 [58%]), female (1023 [65%]), licensed to practice in 2000 or later (940 [61%]), and spent fewer than 40 hours per week in direct patient care (1083 [71%]). Most viewed open notes positively, agreeing they are a good idea (1182 participants [74%]); of 1314 clinicians who were aware that patients were reading their notes, 965 (74%) agreed that open notes were useful for engaging patients. In all, 798 clinicians (61%) would recommend the practice to colleagues. A total of 292 physicians (37%) reported spending more time on documentation, and many reported specific changes in the way they write their notes, the most frequent of which related to use of language that could be perceived as critical of the patient (422 respondents [58%]). Most physicians (1234 [78%]) favored being able to determine readily that their notes had been read by their patients.

**Conclusions and Relevance:**

In this survey of clinicians in a wide range of specialties who had several years of experience offering their patients ready access to their notes, more than two-thirds supported this new practice. Even among subgroups of clinicians who were less enthusiastic, most endorsed the idea of sharing notes and believed the practice could be helpful for engaging patients more actively in their care.

## Key Points

**Question:**

How do clinicians with experience in sharing notes with patients view this new practice?

**Findings:**

In this web-based survey study of 1628 clinicians, most viewed note sharing positively (74% agreed that it is a good idea and 74% viewed shared notes as useful for engaging patients in their care), and 37% of physicians surveyed reported spending more time in documentation. Physicians with more years in practice and fewer hours spent in patient care had more positive opinions overall.

**Meaning:**

Findings from this large survey across specialties in institutions with a history of note sharing suggest few drawbacks for clinicians as they prepare for this rapidly evolving change in practice.

## Teaser

This survey study assesses views and experiences with sharing clinical notes (open notes) with patients among several types of clinician.

## Introduction

A decade ago, the notion of offering patients ready access to their clinical notes (open notes) was a fringe idea. Today, the debate over transparency in health care has taken center stage and has become a pressing legislative and regulatory issue.^1,2^ The 21st Century Cures Act of 2016 requires that patients be given electronic access to the information in their medical records, and recent regulations from the Office of the National Coordinator for Health Information Technology substantially expand the type of information that must be both easily accessible to patients and readily exchanged among clinicians in electronic form. Coupled with the new price transparency required by the Centers for Medicare and Medicaid Services,^3^ patients will soon have easier access to far more detailed information about their care.

In 2012, findings were published^4^ from a pilot project examining the effects of sharing clinical notes with patients receiving primary care in 3 health care systems. The findings suggested patients derived potentially important benefits from reading their notes, and few clinicians reported negative effects on workflow or documentation practices. Thereafter, the 3 systems spread the availability of these notes through virtually all their ambulatory care practices, and recent surveys of more than 20 000 patients at the 3 sites, along with other research conducted in private health care organizations and the Veterans Administration, have shown similar results.^5,6,7,8,9,10^ The practice of note sharing has spread, and at the beginning of 2020, more than 44 million patients at more than 200 US health care organizations have access to their notes through patient portals.^11^

The early reports from physicians on the effects of sharing clinical notes with patients via secure online portals were based on a limited sample of volunteering primary care physicians (PCPs) who were given the opportunity to exclude some of their patients.^4^ Whether these positive results would hold across clinicians of different types who share notes with virtually all their patients is unknown. Negative impacts might emerge when a wide variety of patients are accessing and reading their notes across specialties over time.

In the pilot organizations, virtually all the clinicians, including many in mental illness specialties, have now been sharing their office notes for 4 years or more. Based on our experiences working with health care organizations to implement open notes, we hypothesized that both PCPs and specialists would hold positive views about sharing notes. We expected that PCPs, younger physicians, and female physicians would be more positive overall, and that some would report changes in their documentation practices. To examine these issues, we surveyed a broad array of clinicians in all specialties practicing at our original pilot sites, including physicians, advanced practice nurses (APNs), physician assistants (PAs), registered nurses, therapists, and other clinicians. We examined their experiences with and perceptions about sharing notes with patients and their reports of the effects of this transparency on their documentation practices.

## Methods

### Setting

We conducted a web-based survey of clinicians in hospital-based offices and community practices at 3 health systems: Beth Israel Deaconess Medical Center (BIDMC) (Boston, Massachusetts), Geisinger (Pennsylvania), and University of Washington Medicine (UW) (Seattle). At each site, notes are shared by almost all outpatient clinicians, including PCPs, specialist physicians, APNs, PAs, therapists, and others. The institutional review boards at BIDMC, Geisinger, and UW approved the survey and study protocol at their respective sites. Each waived the requirement for informed consent, as answering the survey was deemed to be implied consent. Reporting of this study follows the American Association for Public Opinion Research (AAPOR) reporting guideline.

### Participants

The survey included clinicians in multispecialty outpatient care practices: at the hospital and 6 affiliated sites at BIDMC, at 3 hospitals and 9 freestanding clinics at UW, and at 7 hospitals and 53 outlying practices at Geisinger. We contacted all clinicians who had at least 1 visit note opened by a patient in the year prior to the survey.

### Constructing the Questionnaire

This survey draws heavily on the original pilot questionnaire and includes additional questions regarding clinician characteristics and changes in documentation practices.^4^ Clinicians who reported they were not aware that patients were reading their notes were asked only if they agreed or disagreed with the statement “making notes available to patients online is a good idea,” whether they would like an indicator in the electronic health record showing a note had been read, and demographic questions. The questionnaire is available in the eAppendix in the Supplement.

### Conducting the Survey

We sent invitations to clinicians’ institutional email addresses between May and August 2018 using REDCap (Vanderbilt), an online, public use secure data management package. Each invitation contained the clinician’s unique study identification number embedded in a link to the survey. We sent clinicians up to 3 reminders 1 week apart if they had not completed the survey. Each site offered clinicians a modest incentive by lottery, available to those who submitted completed surveys. Participating clinicians at the BIDMC and UW could win 1 of 5 $500 prizes (paid as a check at BIDMC and gift card at UW), and at Geisinger they were eligible to win 1 of 25 $100 checks. Clinicians completed the survey from May 21, 2018, to August 31, 2018.

### Statistical Analysis

We categorized clinicians who completed the survey as PCPs, specialist physicians, APNs or PAs, or other clinicians, based either on survey responses or administrative data when the response about professional role was missing. Respondent sex was taken from administrative data. All items reported in this analysis had less than 4% missing responses. Using descriptive statistics, we first compared respondents with nonrespondents using variables from the sampling file (sex and age). Responses using a 4-point agree-disagree scale were collapsed into 2 categories: agree or somewhat agree and disagree or somewhat disagree. We dichotomized survey items addressing frequency: daily, weekly, or monthly and less than monthly or never. Because documentation burden for physicians is such a pressing and important topic, we restricted our analysis of how documentation practices may change owing to open notes to physicians. We used the χ^2^ of independence test to test for differences among clinician groups. The threshold for statistical significance was set at 2-sided *P* < .05. We conducted all the statistical analyses at BIDMC using SAS software version 9.4 (SAS Institute Inc).

## Results

### Participants

We sent invitations to 6064 clinicians, and 1628 responded (response rate = 27%, using the Response Rate Calculation 2 of the AAPOR guideline) (eFigure in the Supplement).^12^ Respondents were more likely than nonrespondents to be female (65% vs 55%) and to be younger (mean [SD] age, 42.1 [12.6] vs 44.9 [12.7] years) (eTable in the Supplement). The majority of respondents were physicians (951 [58%]), female (1023 [65%]), licensed to practice in 2000 or later (940 [61%]), and spent fewer than 40 hours per week in direct patient care (1083 [71%]) (Table 1).

**Table 1.** Respondent Characteristics

Characteristic	No. (%)					*P* value^b^
	Total (N = 1628)	PCP (n = 297)	Specialist (n = 654)	APN or PA (n = 212)	Other (n = 440)^a^	
In what year were you first licensed to practice?						
Before year 2000	591 (39)	120 (42)	202 (32)	54 (26)	215 (51)	<.001
Year 2000 or after	940 (61)	165 (58)	420 (68)	152 (74)	203 (49)	
On average, how many hours per week do you see patients?						
<40 h	1083 (71)	205 (72)	394 (63)	132 (64)	352 (84)	<.001
≥40 h	451 (29)	79 (28)	232 (37)	74 (36)	66 (16)	
Sex^c^						
Female	1023 (65)	184 (62)	293 (45)	186 (88)	360 (86)	<.001
Male	548 (35)	113 (38)	352 (55)	26 (13)	57 (14)	

Abbreviations: APN, advanced practice nurse; PA, physician assistant; PCP, primary care physician.

^a^ The category of other included 206 registered nurses, 84 therapists, 63 mental health clinicians, and 87 other clinicians.

^b^ A χ^2^ test was used for between-group differences.

^c^ Sex was determined from administrative data and was missing for 32 respondents.

### Perceptions and Experiences of All Clinicians

Seventy-four percent of clinicians (1182) agreed that making notes available to patients is a good idea, and 78% (1234) reported they would find it helpful to have an electronic health record indicator showing whether a patient had read a note (Figure). Among the 1314 clinicians (82%) who were aware that patients were reading their notes, 74% (965) agreed that open notes are a useful tool for engaging patients in their care, and 61% (798) would recommend open notes to colleagues at other institutions (Table 2).

**Figure.**
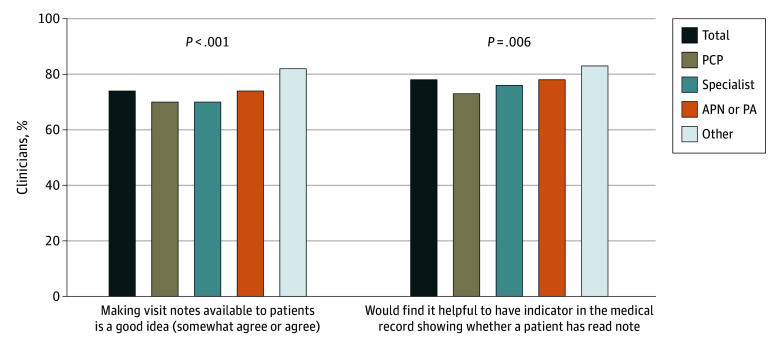
Survey Results Clinicians were asked whether they agreed with the statement “Making visit notes available to patients is a good idea.” They were also asked, “Would you find it helpful to have an indictor in the medical record showing whether a patient has read a note?” APN indicates advanced practice nurse; PA, physician assistant; and PCP, primary care physician.

**Table 2.** Perceptions of and Experiences With Open Notes of Clinicians Aware That Patients Were Reading Their Notes

Perception or experience	No. (%)				*P* value^b^
	Total	Physician	APN or PA	Other^a^	
In general, open notes are a useful tool for engaging patients in their care					
Agree or somewhat agree	965 (74)	552 (69)	133 (75)	280 (83)	<.001
Disagree or somewhat disagree	348 (26)	248 (31)	44 (25)	56 (17)	
Would you, or would you not, recommend open notes to your colleagues at other institutions?					
Would recommend	798 (61)	457 (57)	107 (60)	234 (70)	<.001
Would not recommend	514 (39)	343 (43)	70 (40)	101 (30)	
In the last month, did you encourage any of your patients to read their notes?					
Yes	330 (25)	180 (22)	42 (24)	108 (32)	.003
No	983 (75)	620 (78)	135 (76)	228 (68)	
In the past 12 mo, during office visits, how often did a patient bring up something about a note that you had written?					
Daily, weekly, or monthly (1-3 times/mo)	232 (18)	152 (19)	29 (17)	51 (15)	.33
Less than monthly or never	1067 (82)	644 (81)	145 (83)	278 (85)	
In the past 12 mo, outside of office visits, how often did a patient contact you or your practice with questions about your note?					
Daily, weekly, or monthly (1-3 times/mo)	187 (14)	131 (16)	24 (14)	32 (10)	.01
Less than monthly or never	1112 (86)	665 (84)	150 (86)	297 (90)	
Because of open notes do you spend					
More time writing notes	463 (36)	292 (37)	57 (33)	114 (36)	.002
No change	794 (62)	485 (62)	114 (66)	195 (61)	
Less time writing notes	14 (1)	4 (1)	0	10 (3)	
Has open notes affected the value of your notes for other clinicians					
More valuable	70 (6)	26 (3)	14 (8)	30 (9)	<.001
No change	966 (76)	586 (75)	140 (82)	240 (75)	
Less valuable	234 (18)	168 (22)	17 (10)	49 (15)	

Abbreviations: APN, advanced practice nurses; PA, physician assistants.

^a^ The category of other included 206 registered nurses, 84 therapists, 63 mental health clinicians, and 87 other clinicians.

^b^ A χ^2^ test was used for between-group differences.

Twenty-five percent of clinicians (330) reported encouraging patients to read their notes, 18% (232) said patients mentioned notes during visits at least monthly, and 14% (187) reported patients contacting the office about their notes outside of visits at least monthly (Table 2). Thirty-six percent of clinicians (463) reported spending more time writing their notes because of open notes, while 63% (808) reported no change or spending less time. Eighteen percent of clinicians (234) felt that because of changes due to open notes, their notes became less valuable to other clinicians. While most clinicians said open notes had no effect on the value of their notes, physicians reported that sharing notes with patients reduced the value of their documentation more often than other clinicians (physicians, 22%; APN or PA, 10%; other, 15%; *P* < .001).

### Physician Perceptions, Experiences, and Characteristics

Most physician respondents held positive views about open notes. However, there were significant differences by physician characteristics. Primary care physicians more often stated that they would recommend the practice to colleagues (PCP, 64% vs specialist, 54%; *P* = .008) (Table 3). Compared with those licensed earlier, fewer physicians licensed to practice since 2000 said that they would recommend open notes to colleagues at other institutions (before 2000, 65% vs 2000 or later, 53%; *P* = .001). Compared with those who spent less than 40 hours per week on patient care, physicians spending at least 40 hours per week more often agreed that making notes available to patients was a good idea (<40 hours, 74% vs ≥40 hours, 64%; *P* < .001), that open notes were useful for engaging patients in their care (<40 hours, 72% vs ≥40 hours, 63%; *P* = .01), and that they would recommend open notes to colleagues in other institutions (<40 hours, 61% vs ≥40 hours, 50%; *P* = .004).

**Table 3.** Physicians’ Perceptions of and Experiences With Open Notes

Perception or experience	Total, No. (%)	Physician type			Sex			Hours in patient care			License year		
		PCP, No. (%)	Specialist, No. (%)	*P* value	Female, No. (%)	Male, No. (%)	*P* value	<40 h/wk, No. (%)	≥40 h/wk, No. (%)	*P* value	<2000, No. (%)	≥2000, No. (%)	*P* value
Agree or somewhat agree with the statement, “Making visit notes available is a good idea”^a^	667 (71)	209 (70)	458 (70)	.92	326 (68)	335 (72)	.21	443 (74)	198 (64)	<.001	237 (74)	402 (69)	.12
Agree or somewhat agree with the statement, “Open notes are a useful tool for engaging patients in their care”	552 (69)	184 (74)	368 (67)	.05	269 (69)	278 (70)	.75	375 (72)	153 (63)	.01	206 (71)	323 (68)	.28
Answered yes to the question, “Would you recommend open notes your colleagues at other institutions?”	457 (57)	160 (64)	297 (54)	.008	219 (56)	234 (59)	.43	319 (61)	122 (50)	.004	188 (65)	254 (53)	.001
Because of open notes													
Spend more time writing notes	292 (37)	93 (38)	199 (37)	.15	170 (44)	119 (31)	<.001	190 (36)	95 (39)	.57	88 (30)	197 (41)	.009
Spend less time writing notes	4 (1)	3 (1)	1 (0)		2 (0)	2 (0)		330 (63)	147 (60)		200 (69)	278 (58)	
No change	485 (62)	148 (61)	337 (63)		211 (55)	268 (69)		2 (0)	2 (1)		1 (0)	3 (1)	
Effect of open notes on the value of your notes for other clinicians													
More valuable	26 (3)	6 (2)	20 (4)	.56	11 (3)	15 (4)	.69	19 (4)	7 (3)	.31	8 (3)	18 (4)	.09
No change	586 (75)	188 (77)	398 (74)		286 (75)	293 (75)		401 (77)	178 (73)		231 (80)	349 (73)	
Less valuable	168 (22)	50 (20)	118 (22)		85 (22)	81 (21)		102 (20)	59 (24)		50 (17)	111 (23)	

Abbreviation: PCP, primary care physician.

^a^ Includes respondents who were not aware that patients were reading their notes.

A total of 292 physicians (37%) reported spending more time on documentation. More female physicians reported increased time spent on documentation compared with their male counterparts (female, 44% vs male, 31%; *P* < .001), as did those licensed to practice after 2000 compared with those licensed prior to 2000 (2000 or later, 41% vs before 2000, 30%; *P* = .009). More than three-quarters of both PCPs (77%) and specialists (74%) reported that open notes had no effect on the value of their notes for other clinicians. We found no significant differences in beliefs about the effects of open notes on the value of documentation by sex, hours worked, or year of license.

### Open Notes and Documentation of Physician Encounters

Physicians reported that open notes led them to make several changes in the way they document visits (Table 4). In general, primary care physicians and female physicians more often reported making changes, as did those licensed to practice medicine after 2000 compared with those licensed to practice before 2000. The change cited most frequently related to the use of language that could be perceived as critical of the patient (422 physicians [58%]).

**Table 4.** Changes in Note Writing Among Physicians

Changes in note writing	Total	Physician type			Sex			Hours in patient care			License year		
		PCP, No. (%)	Specialist, No. (%)	*P* value	Female, No. (%)	Male, No. (%)	*P* value	<40 h/wk, No. (%)	≥40 h/wk, No. (%)	*P* value	<2000, No. (%)	≥2000, No. (%)	*P* value
Use of language that could be perceived as critical of the patient	422 (58)	145 (63)	277 (56)	.05	232 (64)	186 (52)	.001	282 (58)	133 (59)	.84	134 (52)	281 (61)	.02
How you document sensitive clinical, mental health, or social information	372 (49)	127 (53)	245 (48)	.16	199 (54)	170 (45)	.02	246 (48)	120 (52)	.38	120 (43)	246 (53)	.006
Use of terms such as “noncompliant,” “patient refuses,” and “patient denies”	306 (41)	113 (49)	193 (38)	.006	172 (47)	132 (36)	.002	206 (41)	94 (41)	.83	105 (39)	196 (42)	.40
How you document patients' perspectives, preferences, and concerns	229 (30)	89 (37)	140 (27)	.003	126 (34)	100 (26)	.02	150 (29)	76 (32)	.38	67 (24)	159 (34)	.003
How you document differential diagnosis	176 (23)	63 (26)	113 (21)	.15	93 (25)	81 (21)	.26	115 (22)	56 (23)	.70	54 (19)	117 (25)	.06
Use of partnering or encouraging language	166 (22)	61 (26)	105 (21)	.11	96 (26)	69 (19)	.02	109 (21)	53 (23)	.61	50 (18)	113 (24)	.05
Use of medical jargon or abbreviations	139 (18)	62 (26)	77 (15)	<.001	78 (21)	60 (16)	.07	100 (19)	37 (16)	.26	51 (18)	86 (18)	.97

Abbreviation: PCP, primary care physician.

## Discussion

In our survey of clinicians in a wide range of specialties who had several years of experience offering their patients ready access to their notes, more than two-thirds supported this new practice. Some subgroups of clinicians were less enthusiastic than others, but even among these, most endorsed the idea of sharing notes and believed the practice could be helpful for engaging patients more actively in their care. While slightly more than one-third of clinicians reported spending more time in documentation, most found the practice did not affect their workflow and would recommend it to colleagues at other institutions. Physicians spending fewer than 40 hours per week in direct patient care were more positive about open notes than were those with more practice hours. Contrary to our expectations, physicians with more years in practice were more positive than were those with fewer years of experience. As the health care system moves toward offering patients ready access to clinical notes, our findings overall suggest few drawbacks for clinicians and health care organizations worried about being overwhelmed by this move toward transparent communication.

The results of our survey indicated that older physicians were more comfortable with open notes. We can only speculate on the reasons for this. More years in practice bring more established relationships, and perhaps greater appreciation for the importance of communication, along with greater confidence in listening and note-writing skills. Conversely, young physicians may feel more stress, competing priorities, or anxiety about building trust with their patients.

Our findings suggest that clinicians are generally positive about open notes; however, some responses indicate this result should be interpreted with caution and explored further. Approximately one-third of clinicians report that because of open notes they are spending at least some additional time in documentation. Even if the actual increase in time is minimal, such perceptions are important. While we do not have independent confirmation of actual increased time spent, these reports may signal additional logistical, cognitive, or linguistic effort clinicians perceive when they write shared notes. Many physicians reported modifying the way they document visits, reporting, in particular, changing their use of critical language and sensitive information. Other research suggests that some clinicians may simplify their language, while others make it more complex.^13,14^ Given the current focus on documentation burden, we need to learn far more about whether and how note sharing is changing documentation practices.

Physicians with greater patient care responsibilities had less positive perceptions of open notes, but we found no differences in time spent on documentation or how notes were written between these physicians and those seeing patients for fewer than 40 hours per week. One possible explanation is that while note sharing may not be changing workflow substantially, busy clinicians may view it as simply one more thing to think about during the day. Additionally, innovation fatigue may play a role.^15^ Clinicians, weary from the pace of change in health care, may simply be uninterested in any new initiative.

More female physicians reported making changes to their notes and spending more time on documentation compared with their male counterparts. Prior research has found that female physicians show more empathy toward patients, ask more questions, and spend more time talking with them than their male counterparts.^16,17^ Increased time and changes in documentation may reflect the relational nature of the way female physicians practice. Research also suggests that female physicians are at increased risk for burnout. Whether for female or male physicians, it is critical that health care organizations provide adequate support to ensure that note sharing does not increase the challenges of documentation in a way that leads to greater burnout.^18^

Reports of more time in documentation should also be assessed in the context of patient reports of the impact of reading their notes. Surveys find that patients overwhelmingly want access to their notes and report benefits from reading them that may have important clinical implications.^7^ They indicate that reading notes improves their trust, helps them feel more in control of their care, is important in helping them to understand what their clinicians are thinking, and helps them adhere to treatment plans and medications more effectively. Patients also state that the availability of notes will affect their future choice of a health care provider.^7,19^ Moreover, this improved trust and associated relational benefits may accrue to both patients and clinicians, resulting in stronger relationships.^19,20,21^

Furthermore, more than 3 out of 4 clinicians felt that knowing which of their patients had reviewed their notes would be helpful. To our knowledge, none of the major electronic health record vendors offer such functionality routinely. Indeed, few are able to calculate the percentage of notes patients read. Not knowing whether a patient has read a note may explain why few physicians report discussing them with patients. Feedback loops and measurements that are helpful to both patients and clinicians remain works in progress.

### Limitations

Our study has a number of limitations. First, we surveyed clinicians in 3 health care organizations that began sharing notes in 2010, limiting the generalizability of our findings. Clinicians in other types of organizations and those without a long history of note sharing may have different opinions and experiences. Second, our survey response rate was modest, and those who responded may have differed from nonresponders in attitudes and experiences. It is well known that survey response rates overall are declining, and surveys of physicians are no exception.^19,20^ However, our response rate was not markedly different from, and in some cases was better than, other physician surveys conducted online.^22,23,24,25,26,27^ Furthermore, a low response rate is not necessarily an indication of response bias,^28,29^ particularly for physician respondents,^30^ but we cannot eliminate the possibility that our respondents may differ in important ways from those who did not respond to the survey. They may have been systematically more or less enthusiastic about open notes, thereby creating response bias. However, without more information on the attitudes and experiences of nonrespondents, we can only speculate on the direction this bias might take. Third, as with most survey research, we relied on the accuracy of respondent self-report. In particular, we have not verified respondents’ reports of changes in the amount of time spent or changes to documentation. It is possible that their responses were affected by other unmeasured factors related to their work. In addition, while we drew heavily from a previously developed questionnaire, we did not conduct formal validity and reliability testing for the survey instrument.^4^

## Conclusions

In 1996, the Health Insurance Portability and Accountability Act (HIPAA) gave patients access to the information in their medical records.^31^ In the years since, the widespread adoption of electronic health records and patient portals has made it technically easy for clinicians and health care organizations to offer patients digital access to their medical records. Most people in the US can now use patient portals to make appointments, view test results, request medication refills, and send messages to a clinician, but access to notes has lagged.^32^ Nevertheless, many individuals now have access to their notes, and the 21st Century Cures Act and regulations will make note sharing more common in the coming years.^1,2^

Open notes may help clinicians, patients, and families improve care by moving toward more open communication and partnership. Findings from this large survey across specialties in institutions with a history of note sharing suggest few drawbacks for clinicians as they prepare for this change. As they explore this new practice further, the next challenge comes in providing adequate education and support to patients, families, clinicians, and health systems. If organizations do not prioritize this work of education and culture change, patients may not know that clinicians write notes, many more will not realize that they might benefit from reading them, and we may miss the opportunity to capitalize on the benefits note sharing may bring. Establishing transparency within the fabric of practice is progressing, but considerable work lies ahead before it becomes a new standard of care.

## Notes

Supplement. **eAppendix.** OpenNotes Clinician Survey 2018 **eFigure.** Survey Sample and Respondents **eTable.** Comparing Non-respondents to Respondents

## References

[1] Office of the National Coordinator for Health Information Technology. 21st Century Cures Act: Interoperability, Information Blocking, and the ONC Health IT Certification Program. Published 2020. Accessed March 9, 2020. https://www.healthit.gov/cerus/sites/cerus/files/2020-03/ONC_Cures_Act_Final_Rule_03092020.pdf

[2] Office of the National Coordinator for Health Information Technology. United States Core Data for Interoperability. Published 2020. Accessed March 9, 2020. https://www.healthit.gov/isa/united-states-core-data-interoperability-uscdi

[3] Centers for Medicare and Medicaid Services. CMS advances interoperability and patient access to health data through new proposals. Published February 8, 2019. Accessed September 9, 2019. https://www.cms.gov/newsroom/fact-sheets/cms-advances-interoperability-patient-access-health-data-through-new-proposals

[4] Delbanco T, Walker J, Bell SK, . Inviting patients to read their doctors’ notes: a quasi-experimental study and a look ahead. Ann Intern Med. 2012;157(7):461-470. doi:10.7326/0003-4819-157-7-201210020-00002 23027317 PMC3908866

[5] Dobscha SK, Denneson LM, Jacobson LE, Williams HB, Cromer R, Woods S. VA mental health clinician experiences and attitudes toward OpenNotes. Gen Hosp Psychiatry. 2016;38:89-93. doi:10.1016/j.genhosppsych.2015.08.001 26380876

[6] Woods SS, Schwartz E, Tuepker A, . Patient experiences with full electronic access to health records and clinical notes through the My HealtheVet Personal Health Record Pilot: qualitative study. J Med Internet Res. 2013;15(3):e65. doi:10.2196/jmir.2356 23535584 PMC3636169

[7] Walker J, Leveille S, Bell S, . OpenNotes after 7 years: patient experiences with ongoing access to their clinicians’ outpatient visit notes. J Med Internet Res. 2019;21(5):e13876. doi:10.2196/13876 31066717 PMC6526690

[8] Mishra VK, Hoyt RE, Wolver SE, Yoshihashi A, Banas C. Qualitative and quantitative analysis of patients’ perceptions of the patient portal experience with OpenNotes. Appl Clin Inform. 2019;10(1):10-18. doi:10.1055/s-0038-1676588 30602196 PMC6327733

[9] Huang JS, Yueh R, Ma S, Cruz R, Bauman L, Choi LJ. Adolescents’ and young adults’ satisfaction with and understanding of medical notes from a pediatric gastroenterology practice: a cross-sectional cohort study. J Pediatr. 2019;215:264-266. doi:10.1016/j.jpeds.2019.06.052 31377044

[10] Giannouli V. Giving doctors’ daily progress notes to hospitalized patients and families: a reflection. Am J Med Qual. 2017;32(4):459. doi:10.1177/1062860617699698 28665711

[11] OpenNotes. OpenNotes homepage. Accessed November 26, 2019. https://www.opennotes.org/

[12] American Association for Public Opinion Research. Standard definitions: final dispositions of case codes and outcome rates for surveys. 9th Edition. Published 2016. Accessed January 14, 2020. https://www.aapor.org/AAPOR_Main/media/publications/Standard-Definitions20169theditionfinal.pdf

[13] Klein JW, Jackson SL, Bell SK, . Your patient is now reading your note: opportunities, problems, and prospects. Am J Med. 2016;129(10):1018-1021. doi:10.1016/j.amjmed.2016.05.015 27288854 PMC7098183

[14] Rahimian M, Warner JL, Jain SK, Davis RB, Zerillo JA, Joyce RM. Significant and distinctive *n*-grams in oncology notes: a text-mining method to analyze the effect of OpenNotes on clinical documentation. JCO Clin Cancer Inform. 2019;3:1-9. doi:10.1200/CCI.19.00012 31184919 PMC6873977

[15] Chung GH, Choi JN, Du J. Tired of innovations? learned helplessness and fatigue in the context of continuous streams of innovation implementation. J Organ Behav. 2017;38(7):1130-1148. doi:10.1002/job.2191

[16] Howick J, Steinkopf L, Ulyte A, Roberts N, Meissner K. How empathic is your healthcare practitioner? a systematic review and meta-analysis of patient surveys. BMC Med Educ. 2017;17(1):136. doi:10.1186/s12909-017-0967-3 28823250 PMC5563892

[17] Roter DL, Hall JA. Physician gender and patient-centered communication: a critical review of empirical research. Annu Rev Public Health. 2004;25(1):497-519. doi:10.1146/annurev.publhealth.25.101802.123134 15015932

[18] Puffer JC, Knight HC, O’Neill TR, . Prevalence of burnout in board certified family physicians. J Am Board Fam Med. 2017;30(2):125-126. doi:10.3122/jabfm.2017.02.160295 28379817

[19] Bell SK, Mejilla R, Anselmo M, . When doctors share visit notes with patients: a study of patient and doctor perceptions of documentation errors, safety opportunities and the patient-doctor relationship. BMJ Qual Saf. 2017;26(4):262-270. doi:10.1136/bmjqs-2015-004697 27193032 PMC7255406

[20] DesRoches CM, Bell SK, Dong Z, . Patients managing medications and reading their visit notes: a survey of OpenNotes participants. Ann Intern Med. 2019;171(1):69-71. doi:10.7326/M18-3197 31132794

[21] Bell SK, Folcarelli P, Fossa A, . Tackling ambulatory safety risks through patient engagement: what 10,000 patients and families say about safety-related knowledge, behaviors, and attitudes after reading visit notes. J Patient Saf. 2018;00(00):1-9. doi:10.1097/PTS.0000000000000494 29781979

[22] Pew Research Center. Collecting survey data. Accessed September 26, 2019. https://www.pewresearch.org/methods/u-s-survey-research/collecting-survey-data/#the-problem-of-declining-response-rates

[23] Wiebe ER, Kaczorowski J, MacKay J. Why are response rates in clinician surveys declining? Can Fam Physician. 2012;58(4):e225-e228. 22611609 PMC3325475

[24] Taylor T, Scott A. Do physicians prefer to complete online or mail surveys? findings from a national longitudinal survey [published online November 1, 2018]. Eval Health Prof. doi:10.1177/0163278718807744 30384770

[25] Sebo P, Maisonneuve H, Cerutti B, Fournier JP, Senn N, Haller DM. Rates, delays, and completeness of general practitioners’ responses to a postal versus web-based survey: a randomized trial. J Med Internet Res. 2017;19(3):e83. doi:10.2196/jmir.6308 28330830 PMC5382256

[26] Cook C, Heath F, Thompson RL. A meta-analysis of response rates in web- or internet-based surveys. Educ Psychol Meas. 2000;60(6):821-836. doi:10.1177/00131640021970934

[27] Blackstock OJ, Moore BA, Berkenblit GV, . A cross-sectional online survey of HIV pre-exposure prophylaxis adoption among primary care physicians. J Gen Intern Med. 2017;32(1):62-70. doi:10.1007/s11606-016-3903-z 27778215 PMC5215171

[28] Keeter S, Kennedy C, Dimock M, Best J, Craighill P. Gauging the impact of growing nonresponse on estimates from a national RDD telephone survey. Public Opin Q. 2006;70(5):759-779. doi:10.1093/poq/nfl035

[29] Curtin R, Presser S, Singer E. The effects of response rate changes on the index of consumer sentiment. Public Opin Q. 2000;64(4):413-428. doi:10.1086/318638 11171024

[30] Kellerman SE, Herold J. Physician response to surveys: a review of the literature. Am J Prev Med. 2001;20(1):61-67. doi:10.1016/S0749-3797(00)00258-0 11137777

[31] U.S. Department of Health and Human Services. Summary of the HIPAA privacy rule. Published 2019. Accessed November 13, 2019. https://www.hhs.gov/hipaa/for-professionals/privacy/laws-regulations/index.html

[32] Patel V, Johnson C; The Office of the National Coordinator for Health Information Technology. Individuals’ use of online medical records and technology for health needs. Published 2018. Accessed July 29, 2019. https://www.healthit.gov/sites/default/files/page/2018-03/HINTS-2017-Consumer-Data-Brief-3.21.18.pdf

