# Supplementary material for: The Views and Experiences of Clinicians Sharing Medical Record Notes With Patients
Source: JAMA Netw Open. 2020 Mar 27;3(3):e201753. doi: 10.1001/jamanetworkopen.2020.1753 (PMC12124735; doi:10.1001/jamanetworkopen.2020.1753)
Supplement: Supplement. — eAppendix. OpenNotes Clinician Survey 2018 eFigure. Survey Sample and Respondents eTable. Comparing Non-respondents to Respondents [file jamanetwopen-e201753-s001.pdf]

Supplementary Online Content

DesRoches CM, Leveille S, Bell SK, et al. The views and experiences of clinicians sharing medical record notes with patients. *JAMA Netw Open*. 2020;3(3):e201753. doi:10.1001/jamanetworkopen.2020.1753

eAppendix. OpenNotes Clinician Survey 2018

eFigure. Survey Sample and Respondents

eTable. Comparing Non-respondents to Respondents

This supplementary material has been provided by the authors to give readers additional information about their work.

© 2020 DesRoches CM et al. *JAMA Network Open.*

eAppendix: OpenNotes® Clinician Survey 2018

PART I

1. (Aware)
Before getting this survey, were you aware that your patients are reading your visit notes?

☐ Yes

☐ No → online_good_idea → indicator_ note → Part 4 to end

2. (Online_good_idea)
In general, making visit notes available to patients online ("open notes") is a good idea.

☐ Disagree

☐ Somewhat disagree

☐ Somewhat agree

☐ Agree

3. (Useful_communication)
In general, open notes are a useful tool for engaging patients in their care.

☐ Disagree

☐ Somewhat disagree

☐ Somewhat agree

☐ Agree

4. (Recommend)
Would you, or would you not, recommend open notes to your colleagues at other institutions?

☐ Yes, I would recommend
☐ No, I would not recommend

© 2020 DesRoches CM et al. *JAMA Network Open.*

5. (Encourage)
In the last month, did you encourage any of your patients to read their notes?

☐ Yes

☐ No

6. (Brought_up note)
In the last 12 months, *during* office visits how often did a patient bring up something about a note that you had written?

☐ Daily, almost daily

☐ Weekly

☐ Monthly (1-3 times/month)

☐ Less than monthly

☐ Never

7. (Contact_ questions)
In the last 12 months, outside of office visits, how often did a patient contact you or your practice with questions about your notes?

☐ Daily, almost daily

☐ Weekly

☐ Monthly (1-3 times/month)

☐ Less than monthly

☐ Never to my knowledge

8. (Indicator_ note)
Would you find it helpful to have an indicator in the medical record showing whether a patient has read a note?

☐ Yes

☐ No

© 2020 DesRoches CM et al. *JAMA Network Open.*

PART 2: IMPACT OF OPEN NOTES ON YOUR PATIENTS

In general, your patients who read their visit notes:

9. (Better_prepared)
Are better prepared for visits.

- ☐ Disagree
- ☐ Somewhat disagree
- ☐ Somewhat agree
- ☐ Agree
- ☐ Don't know

10. (Medications)
Are more likely to talk about their medications during an office visit.

- ☐ Disagree
- ☐ Somewhat disagree
- ☐ Somewhat agree
- ☐ Agree
- ☐ Don't know

11. (Confusing)

Find the notes to be more confusing than helpful.

- ☐ Disagree
- ☐ Somewhat disagree
- ☐ Somewhat agree
- ☐ Agree
- ☐ Don't know

© 2020 DesRoches CM et al. *JAMA Network Open.*

12. (Offended)
In the last 12 months, how many of your patients indicated they were offended by something they read in a note?

☐ None

☐ 1-3 patients

☐ 4-10 patients

☐ 11 or more patients

13. (Importance_errors)
Have any of your patients ever notified you of errors in your notes that you thought were important?

☐ Yes

☐ No

If no to importance_errors, skip (Elaborate_errors).

14. (Elaborate_errors)
Please elaborate on the important errors.

15. (Compromised)
Do you know of any instances where care was compromised by a patient reading a note?

☐ Yes

☐ No

If no to compromised skip (Elaborate_compromised).

16. (Elaborate_compromised)

Please describe the situations(s) where care was compromised.

© 2020 DesRoches CM et al. *JAMA Network Open.*

17. (Comment_anecdotes)

Please provide any further comments about the impact of open notes on your patients, including unexpected benefits or issues.

PART 3: IMPACT OF OPEN NOTES ON MY PRACTICE

17. (Time_writing)
Because of open notes, do you spend more or less time writing / dictating / editing your notes?

- ☐ Much more time
- ☐ Somewhat more time
- ☐ No change
- ☐ Somewhat less time
- ☐ Much less time

18. (Between_providers)
Has open notes affected the value of your notes for other clinicians?

- ☐ Much more valuable
- ☐ Somewhat more valuable
- ☐ No change
- ☐ Somewhat less valuable
- ☐ Much less valuable

19. (when_generate)
When do you generate the majority of your notes?

- ☐ During the visit → Q20
- ☐ After the visit → skip Q20

© 2020 DesRoches CM et al. *JAMA Network Open.*

20. (how_generate)
How do you generate the majority of your notes?

☐ Dictation
☐ Type
☐ Scribe

(You _write)
21. Knowing that patients may read your notes, have you changed what you write?

	YES Changed	NO Have not Changed	N/A
**A**. Your use of medical jargon or abbreviations	☐	☐	☐
**B**. Your use of terms such as "non-compliant," "patient refuses," and "patient denies"	☐	☐	☐
**C.** Your use of other language that could be perceived as critical of the patient	☐	☐	☐
**D.** How you document differential diagnosis	☐	☐	☐
**E**. Your use of partnering/encouraging language	☐	☐	☐
**F.** How you document patients' perspectives, preferences, and concerns	☐	☐	☐
**G.** How you document sensitive clinical, mental health, or social information	☐	☐	☐

(If all "no change," skip Elaborate_changes)

22. (Elaborate_changes)
Please provide any further comments on how you have changed what you write.

© 2020 DesRoches CM et al. *JAMA Network Open.*

23. (Additional_comments)
Do you have any additional comments or suggestions related to open notes?

PART 4: ABOUT ME

24. (Clinician_type)
Are you a:

☐ MD / DO

☐ Advanced Practice Nurse

☐ Registered Nurse

☐ Physician Assistant

☐ Therapist (PT, OT, Speech, Rehab, other)

☐ Mental Health Clinician

☐ Other Clinician

Please specify clinician type ___________________

Skip 24 and 26 everyone accept MD/DO

25. (Burnout) MD/DO only
Using your own definition of "burnout," please select one of the statements from the dropdown menu.

☐ I enjoy my work. I have no symptoms of burnout.

☐ I am under stress, and don't always have as much energy as I did, but I don't feel burned out.

☐ I am definitely burning out and have one or more symptoms of burnout, e.g., emotional exhaustion.

☐ The symptoms of burnout that I'm experiencing won't go away. I think about work frustration a lot.

☐ I feel completely burned out. I am at the point where I may need to seek help.

26. (Hours_Week) (optional)

© 2020 DesRoches CM et al. *JAMA Network Open.*

On average, how many hours per week do you see patients? *Please do not include time spent supervising trainees.*

______ hours/week

27. (Consultation_ongoing) (MD/DO optional)
You see patients:

☐ Mainly for consultation or episodic care

☐ Mainly for ongoing care

☐ For both consultation and ongoing care

28. (Licensed) (optional)
In what year were you first licensed to practice?

____

© 2020 DesRoches CM et al. *JAMA Network Open.*

If clinician identifies as Registered Nurse (Q23) they will be prompted to answer additional questions after answering (Q27)

28. What follows is a series of 13 activities roles performed filled by nurses in ambulatory care practice. How do you spend your time? How often do you engage in the following activities?

		Every day	Several times a week	Once a week	1-3 times a month	Never	Unsure or don't know
1.	Assessment and documentation of patient health status and needs	○	○	○	○	○	○
2.	Telephone or email triage	○	○	○	○	○	○
3.	Chronic illness case management and care coordination	○	○	○	○	○	○
4.	Interdisciplinary team meetings	○	○	○	○	○	○
5.	Patient teaching and health coaching	○	○	○	○	○	○
6.	Delegated care for episodic illness using pre-established protocols	○	○	○	○	○	○
7.	Hospital admission and post-discharge management	○	○	○	○	○	○
8.	Medication reconciliation	○	○	○	○	○	○
9.	Staff supervision, precepting students	○	○	○	○	○	○
10.	Responsibilities such as quality improvement, research activities, leadership	○	○	○	○	○	○

© 2020 DesRoches CM et al. *JAMA Network Open.*

11. Assisting with procedures, taking vital signs, collecting specimens, or similar technical tasks	○	○	○	○	○	○
12. Administering medications or injections	○	○	○	○	○	○
13. Enabling operations, such as /equipment, room set up, order supplies, patient transport	○	○	○	○	○	○

29. What proportion of your time do you spend with patients you see in an ongoing way?

☐ < 25%
☐ 25-49%
☐ 50-74%
☐ 75% or more

eFigure: Survey sample and respondents

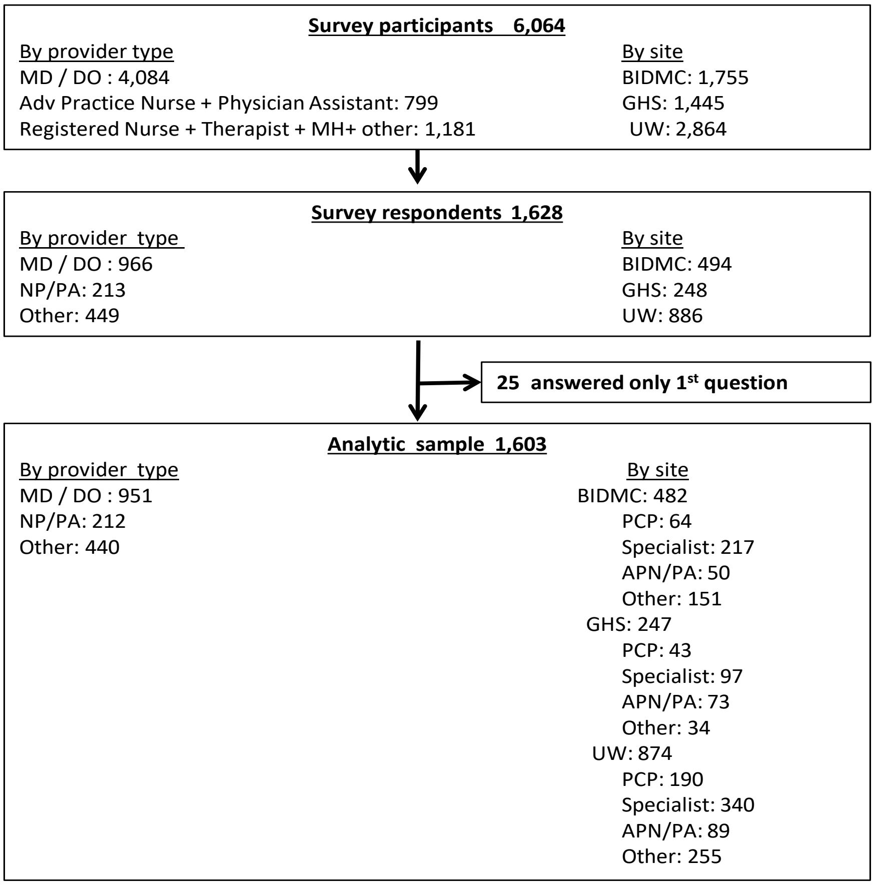

© 2020 DesRoches CM et al. *JAMA Network Open.*

eTable: Comparing non-respondents to respondents

	Total	*Non-respondents*	*Respondents*	*p-value*
	N (%)	N (%)	N (%)	
**Gender**				
Female	3466 (58)	2425 (55)	1041 (65)	<.001
Male	2530 (42)	1973 (45)	557 (35)	
**Mean Age**	--	42.1	44.9	<.001

© 2020 DesRoches CM et al. *JAMA Network Open.*
